# Assessment of the Variability in Influenza A(H1N1) Vaccine Effectiveness Estimates Dependent on Outcome and Methodological Approach

**DOI:** 10.1371/journal.pone.0028743

**Published:** 2011-12-21

**Authors:** Kimberley Kavanagh, Chris Robertson, Jim McMenamin

**Affiliations:** 1 Department of Mathematics and Statistics, University of Strathclyde, Glasgow, United Kingdom; 2 Health Protection Scotland, Glasgow, United Kingdom; 3 International Prevention Research Institute, Lyon, France; Friedrich-Loeffler-Institut, Germany

## Abstract

**Background:**

Estimation of Influenza vaccine effectiveness (VE) varies with study design, clinical outcome considered and statistical methodology used. By estimating VE using differing outcomes and statistical methods on the same cohort of individuals the variability in the estimates produced can be better understood. The Pandemic Influenza Primary Care Reporting (PIPeR) cohort of approximately 193,000 individuals was used to estimate pandemic VE in Scotland during season 2009–10. VE results for three outcomes; influenza related consultations, virological confirmed influenza and death were considered. Use of individualised records allowed all models to be adjusted for age, sex, deprivation, risk status relating to chronic illnesses, seasonal vaccination status and a marker of the individual's propensity to consult. For the consultation and death outcomes, VE was calculated by comparing consultation rates in the unvaccinated and vaccinated groups, adjusted for the listed factors, using both Cox and Poisson regression models. For the consultation outcome, the unvaccinated group was split into individuals before vaccination and those never vaccinated to allow for potential differences in the health seeking behaviour of these groups. For the virology outcome estimates were calculated using a generalised additive logistic regression model. All models were adjusted for time. Vaccine effect was demonstrated for the influenza-like illness consultation outcome using the Cox model (VE = 49% 95% CI (19%, 67%)) with lower estimates from the model splitting the before and never vaccinated groups (VE = 34.2% with 95% CI (−0.5%, 58.9%)). Vaccine effect was also illustrated for overall mortality (VE = 40% (95% CI 18%, 56%)) and a virological confirmed subset of symptomatic individuals (VE = 60% (95% CI −38%, 89%)).

**Conclusions:**

This study illustrates positive point estimates of Influenza VE across methodology and outcome for a single cohort of individuals during season 2009–10. Understanding of potential differences between approaches aids interpretation of VE results in future seasons.

## Introduction

Influenza vaccine effectiveness (VE) requires yearly assessment due to the evolution of the virus and subsequent reformulation of the vaccination. Such changes influence the estimates of VE between seasons. Within a season, differences in estimates for the same vaccination may vary between studies due to methodological differences in the study design, the statistical method employed and the outcome measure used. Generally VE studies fall into one of three designs; case-control (including test negative), cohort or screening. The design of the study dictates the method of analysis used. In the current European context, only observational studies could be used to provide real time influenza VE estimates [1]. In the case of observational cohort studies, the methodology used may be logistic regression, Poisson regression or Cox proportional hazards. For the same outcome these may produce differing estimates.

Estimates may also differ dependent on the clinical outcomes used to measure influenza VE. Typical outcomes are influenza-related GP consultation rates, laboratory confirmed diagnosis, hospitalisation rates and death rates (all or influenza-specific causes). Valenciano *et al.* [2] highlight that the various clinical outcomes used have differing sensitivity and specificity, with low specificity in particular, leading to underestimation of influenza VE estimation.

There have been a number of recent publications on the vaccine effect of the pandemic H1N1v vaccine delivered in the autumn of 2009. All have been on virological confirmed cases of influenza either from GP sentinel schemes using a defined protocol for the swabbing of patients with symptoms or from hospital cases. In England and Scotland a test negative design estimated that the pandemic vaccine had a vaccine effect of 72% (95% confidence interval (CI): 21% to 90%) [3]. A multicentre case control study in seven countries in Europe provided estimates of 71.9% (95% CI 45.6–85.5) [2]. Both of these studies had issues with missing data, particularly the UK study where vaccine status was unknown for many patients; imputation was used in the multicentre study primarily for missing co morbidity information. A Canadian study reported that the vaccine effect was 93% (95% CI 69% to 98%) [4]. In a test negative case control study of hospitalisations for influenza in Castellon, Spain a vaccine effect of 90% (95% CI, 48–100%) was reported [5]. Estimates of VE of 96.8%; (95% CI 95.2–97.9%) in persons aged 14–59 years and 83.3% (95% CI:71.0–90.5%) in those 60 years or older were provided using the screening method on virological samples in Germany [6].

Estimates of seasonal influenza VE in Scotland, using influenza-related consultations within a cohort of patients registered at a number of general practices have been estimated at Health Protection Scotland (HPS) since 2008. For season 2009–2010, we present estimates of pandemic influenza VE calculated using differing statistical methodologies. In addition we present the effect of varying the outcome measure used, firstly in terms of the consultation definition and then considering laboratory confirmed diagnosis and death as end-points. In this way, we aim to understand the variation in VE estimates for the same cohort and identify robust methods for estimating pandemic influenza VE from routinely collected consultation data. Such data has the facility to provide timely estimates throughout the flu season.

## Methods

### Study design

The Pandemic Influenza Primary care Reporting (PIPeR) cohort is based upon 37 practices which were drawn from a sentinel surveillance network of GP practices contributing to the Practice Team Information (PTI) network [7]. In season 2009–2010, PIPeR covered approximately 206,000 patients, around 4% of the Scottish population. Thirty-two of these practices, covering approximately 193,000 patients, gave permission for the extract of pandemic vaccination data alongside the routinely collected data. Seasonal vaccination data are routinely collected but as the pandemic vaccination was new, separate consent for the data extraction had to be undertaken.

The PIPeR cohort contains anonymous individualised records for participants who have at least one year of recorded database history prior to the start of the study, detailing the age, sex, deprivation index, based upon postcode sector and linked to the Carstair's measure [8], and at-risk status for influenza. Daily automated updates provide information on vaccination status (seasonal influenza and pneumococcal polysaccharide vaccination) and consultations corresponding to acute respiratory infections (ARI) and influenza-like illness (ILI). Date of death was recorded and those who died prior to the start date of the study were removed from consideration.

Individuals ‘at risk’ of complications following influenza infection were recorded. The conditions covered were: diabetes, coronary heart disease, chronic liver disease, chronic respiratory disease, chronic liver disease, neurological disorders and immunosuppression. Risk group status was poorly recorded for those over 65 years of age, as they are routinely targeted for vaccination. Risk group status was assigned at the beginning of the cohort and individuals were assumed to remain in that status throughout the period of analysis. The Community Health Index (CHI) is also available at HPS in password protected databases and this is a unique number which can be used to link to other health systems.

### Outcomes

The endpoints considered were consultation rates, laboratory confirmed infection and death.

Three classifications of consultation were considered: influenza-like illness consultations denoted ILI, acute respiratory infection consultations including influenza-like illness denoted ARI, and a combined total number of both consultations, excluding those which are Asthma-related, denoted ILIARI. For each endpoint, the consultation was excluded from the analysis if it occurred within 7 days of the pandemic influenza vaccination to allow for the time required for a protective effect to be established. For seasonal influenza vaccination the conventional exclusion period is 14 days, however studies at the Health Protection Agency and the sensitivity analysis in Hardelid *et al.* [3] suggest 7 days is sufficient for the Influenza A (H1N1v) vaccination.

Among patients who attended the PIPeR practices, a subset of those with clinical symptoms which resembled those associated with influenza like illness, were swabbed as part of the Scottish Sentinel swabbing scheme.

The date of death was extracted from the practice database but there is no information on cause of death. The analysis is based upon dates of death extracted from the practice records up to 30^th^ June 2010. In a sensitivity analysis to investigate delays in recording date of death, the extract was carried on to the end of October 2010 and an additional 2 deaths within the study period were noted.

### Vaccination status and study period

Influenza vaccination was coded as a dichotomous time-dependent variable with recording of both the seasonal and pandemic vaccination in season 2009/2010. The study period is from October 1st 2009, (26^th^ October, 2009 was the date of the first pandemic Influenza A (H1N1v) vaccinations in the UK) and ended on 31^st^ March 2010. Seasonal vaccinations from September 1^st^, 2009 were recorded.

#### Statistical analysis

The analysis differed depending upon the outcome and statistical method used. For consultation outcomes there were potentially multiple events per patient whereas for death and virological status each patient had only one possible event.

There are two time dependent covariates – Seasonal vaccine status and pandemic influenza A (H1N1v) vaccine status and changes to either result in a split of the patients follow up record. Such time dependency makes Cox proportional hazards [9] an appropriate method to use.

The effect of the following covariates which may influence vaccine effectiveness were considered for all outcomes: age group, gender, risk group status (in at least one clinical risk group, yes/no), number of ILIARI consultations in the previous influenza season (0, 1, 2+), seasonal influenza vaccination status in the previous season, seasonal influenza vaccination status in the current season and deprivation represented using quintiles based on the Carstair's index [8]. Estimates of adjusted vaccine effectiveness, using Cox regression were calculated as VE = (1-RR)*100 where the relative risk (RR) is the exponent of the hazard ratio of vaccine status (vaccinated/not vaccinated).

### Consultation outcome

Each new consultation event leads to a new record in the set up for the time dependent cox model. Multiple consultations for the same event on the same day were counted as one consultation however two consultations one day apart were counted as two consultations. An extension to the standard time dependent Cox model, using robust standard errors, to account for clustering of individuals within practices, is also considered.

The Cox model can be recast as a Poisson linear-regression model [10] by aggregating the person time at risk. Time was stratified in weeks and the number of consultation events in each covariate pattern and vaccine status per week was calculated along with the time at risk in that week. In the Poisson model, adjusted vaccine effectiveness is calculated as VE = (1-RR)*100 where RR is the ratio of the consultation rate among those unvaccinated compared to those vaccinated.

The Cox and Poisson models make no distinction between the consultation rates in those never vaccinated relative to the rates in those who are ultimately vaccinated but are prior to vaccination. In an extension of the Poisson model, a patient's exposure to vaccine at any time was recoded as never vaccinated, before vaccination and after vaccination. A comparison of the consultation rates before and after vaccination eliminates some of the effects of confounding variables and the propensity to consult, and provided the temporal trend is modelled appropriately will give an estimate of vaccine effect. In the extended Poisson model, adjusted vaccine effectiveness was calculated as VE = (1-RR)*100 where RR is the ratio of the consultation rate after vaccination compared to the consultation rate before vaccination.

Both models using the Poisson regression framework were adjusted for time by using a factor to differentiate weeks since the start date and so accounting for changes in the background rate of disease in the community.

Finally, the screening method [11] was used to illustrate estimates of VE in a situation where only aggregate data was available. To do so the data was aggregated for each GP practice to give summaries of; the number of consultations in vaccinated individuals, the total number of consultations and the vaccination coverage in that practice. VE is calculated using a generalised linear mixed model where the log odds of a consultation in the vaccinated group is the response, the log odds of the vaccine coverage is the offset and the GP practice is the random effect.

### Death outcome

The death rates among those vaccinated and unvaccinated were compared through a time dependent cox model and a Poisson regression model based upon calculation of the person time at risk for each week throughout the observation period. Adjustment was made for the confounding factors.

### Virology outcome

HPS has data on the results of all virological swab tests for H1N1v influenza during the pandemic season 2009–10. The sample are from all hospital laboratories in Scotland, collected through the Electronic Communication of Surveillance in Scotland (ECOSS) system [12], and the West of Scotland Regional Virus Lab which tests the majority of samples from general practice, including those in the sentinel surveillance scheme. All patients who were tested had acute respiratory symptoms and were tested for clinical reasons. There were 13623 individuals with at least one virological test. Many individuals had multiple tests. The first positive test was selected for those with a positive result and the first test was selected for those with all negative results. Virological data were linked to the cohort on the basis of the community health index number (CHI Number). Laboratory samples without a valid CHI Number were excluded as they could not be linked −11.6% of records

A nested case control analysis was used to estimate vaccine effectiveness by fitting a generalised additive logistic regression model with those who tested negative serving as the controls [13]. As the vaccination was administered to individuals at differing times through the swabbing interval the comparison of the rates of swab positivity among those vaccinated or unvaccinated at the time of swabbing was adjusted for the temporal trends in swab positivity which was modelled by a quadratic trend based upon week of sample collection.

All statistical analysis was conducted using R version 2.12.2.

## Results

### Demographics, vaccine uptake and consultations

The pandemic influenza cohort is composed of 193,034 eligible individuals, 49.8% male, with mean age 40.3 years. Most individuals are in the 15–44 year old age group (41.1%), with 16.0% over 65 and 4.7% under 5 years old (Table 1). Of those under 65, 14.8% are in at least one clinical risk group. The most commonly recorded risk group in those under 65 is chronic respiratory disease (5.4%) followed by chronic heart disease (3.7%) and diabetes (3.2%).

**Table 1:** Demographics of the PIPeR cohort pandemic influenza subset.

		Number			Percentage		
Variable	Level	Cohort	Has at least 1 ILIARI consultation	Has an influenza virology test	Cohort	Has at least 1 ILIARI consultation	Has an influenza virology test
Gender	Female	96954	4922	990	50.2	57.2	57.9
	Male	96080	3690	721	49.8	42.8	42.1
Age Group	<1	235	64	10	0.1	0.7	0.6
	1–4	8779	1807	252	4.5	21.0	14.7
	5–9	20803	1282	288	10.8	14.9	16.8
	15–44	79223	2396	714	41.0	27.8	41.7
	45–64	53016	1768	335	27.5	20.5	19.6
	65–74	16980	722	69	8.8	8.4	4.0
	75+	13998	573	43	7.3	6.7	2.5
Risk Group	No	168189	6935	1346	87.1	80.5	78.7
	Yes	24845	1677	365	12.9	19.5	21.3
Seasonal Flu Vaccine	No	159242	6598	1424	82.5	76.6	83.2
Previous Season	Yes	33792	2014	287	17.5	23.4	16.8
Number of ILIARI consultations in previous year	0	175690	5962	1300	91.0	69.2	76.0
	1	13574	1633	283	7.0	19.0	16.5
	2	2628	582	75	1.4	6.8	4.4
	3+	1142	435	53	0.6	5.1	3.1
Carstairs Quintile (Deprivation)	Low - Q1	21802	1017	137	11.3	11.8	8.0
	Q2	21391	1002	213	11.1	11.6	12.4
	Q3	55571	2343	402	28.8	27.2	23.5
	Q4	54687	2439	431	28.3	28.3	25.2
	High - Q5	38649	1764	514	20.0	20.5	30.0
	Unknown	934	47	14	0.5	0.5	0.8

ILIARI consultation: consultation for Influenza-like illness or acute respiratory infection excluding asthma consultations.

Pandemic and seasonal vaccination uptake by age is summarised in Table 2. For those under 45 in a clinical risk group, H1N1v vaccine uptake is higher than seasonal uptake in the youngest age groups, 0–4 years and 5–14 years. H1N1v uptake is highest in those aged 0–4 years (58.5%) followed by those aged 5–14 years (56.5%). As age increases the differential between seasonal and pandemic vaccination becomes less with similar uptake in the 15–44 year old age group. In the 45–64 year old age group, uptake for the seasonal vaccination exceeds the pandemic uptake. Uptake for those in a clinical risk group is lowest in those aged 15–44 years (39.7%). Overall, H1N1 vaccine uptake is lowest in those aged over 65 (34.8%).

**Table 2:** H1N1 and seasonal vaccine uptake split by clinical risk group and age group.

	In Clinical Risk Group					Not in Clinical Risk Group				
	Number	Pandemic vaccination uptake (%)	95% CI	Seasonal vaccination uptake (%)	95% CI	Number	Pandemic vaccination uptake (%)	95% CI	Seasonal vaccination uptake (%)	95% CI
0–4	258	58.5	(52.4, 64.4)	27.1	(22.1, 32.9)	8756	31.7	(30.7, 32.7)	0.5	(0.3,0.6)
5–14	1516	56.5	(54.0,59.0)	37.5	(35.1, 39.9)	19287	5.1	(4.8,5.4)	0.9	(0.8, 1.0)
15–44	7728	39.7	(38.6, 40.8)	38.1	(37.1, 39.2)	71495	3.3	(3.2,3.4)	1.4	(1.3, 1.5)
45–64	14502	51.3	(50.5, 52.1)	54.6	(53.8, 55.4)	38514	3.2	(3.1, 3.4)	3.4	(3.2, 3.5)
**All Under 65**	24004	48	(47.4,48.6)	47.9	(47.3,48.6)	138052	5.3	(5.2, 5.4)	1.8	(1.7,1.9)
65+*	30978	34.8	(34.3, 35.3)	69.4	(68.9, 69.9)	-	-	-	-	-

**Figure 1:**
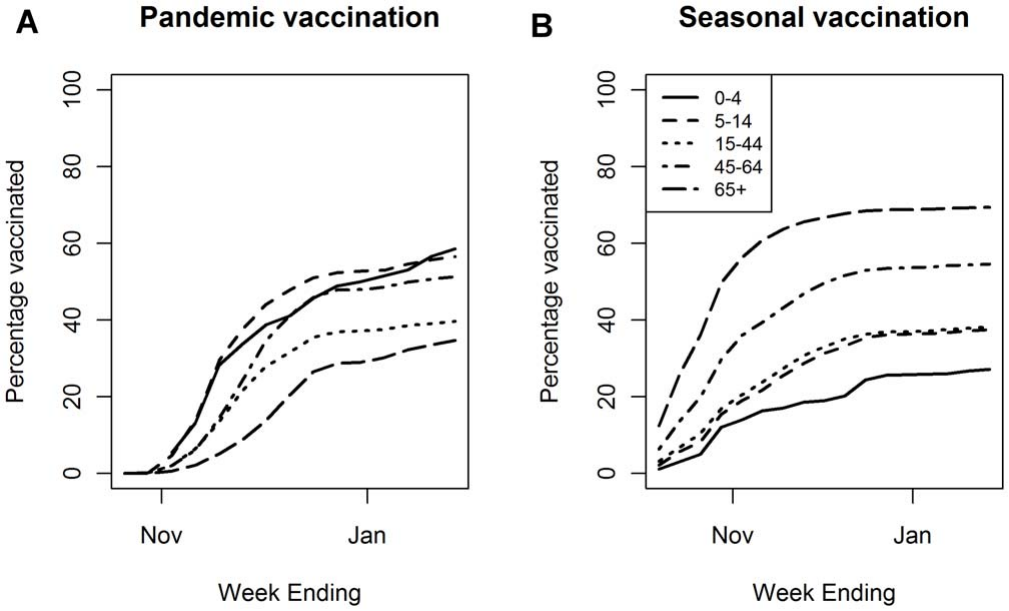
Vaccination administration within the PIPeR cohort by date of administration for all individuals in a risk group. Figure 1A shows pandemic influenza vaccination and Figure 1B seasonal influenza vaccination. All individuals over 65 are automatically “at risk”. Those under 65 are “at risk” if they fall in a clinical risk group.

Figure 1 shows the pandemic and seasonal vaccine uptake over time for “at risk” individuals. Figure 1A illustrates the commencement of the pandemic influenza vaccination in late October 2009 with the majority of the vaccinations administered through November and December 2009. This is in contrast to the seasonal influenza vaccine uptake where the uptake in the elderly had been largely completed by the end of October (Figure 1B). In contrast, a substantial proportion of those in clinical at risk groups only received their seasonal influenza vaccination in the period after1st November.

**Figure 2:**
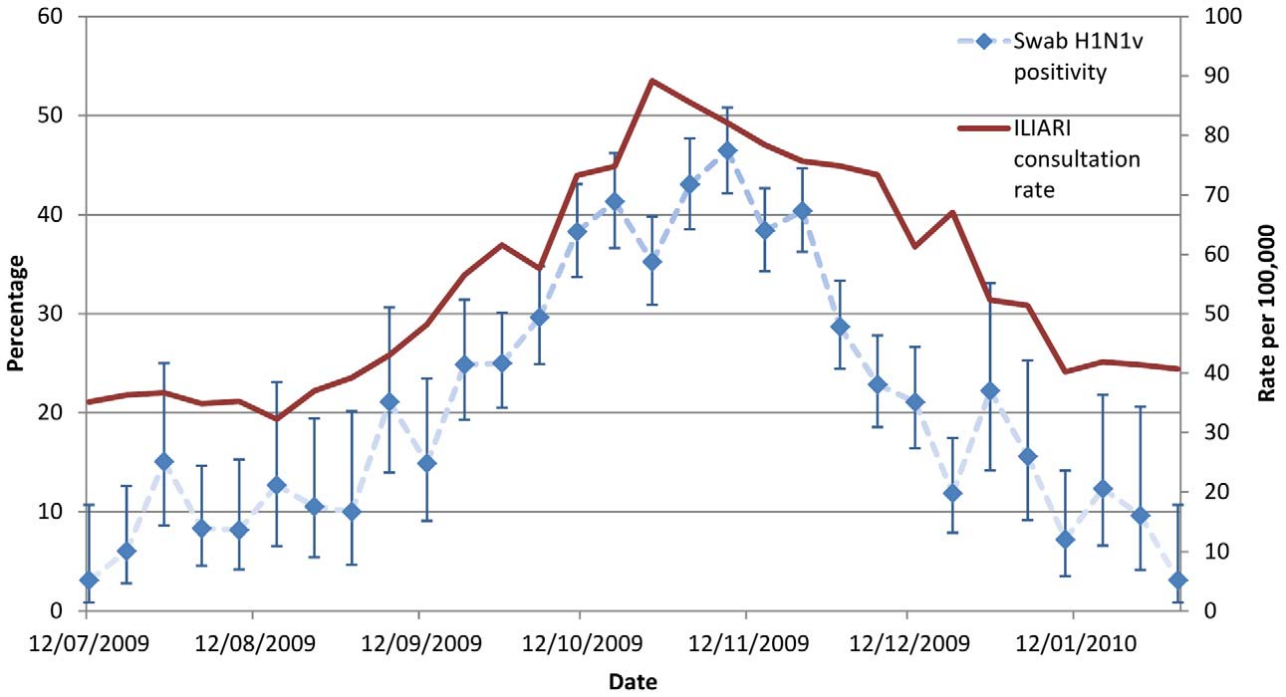
Swab positivity and ILI/ARI GP consultation rates for the week ending 12/7/09 to 31/1/2010.

Figure 2 demonstrates the increased clinical reporting of cases of influenza like illness and other acute respiratory infections reported in the 37 PIPeR GP practices from early July 2009 when routine swabbing of a subset of patients attending with influenza-like illness symptoms began, until the 31 January 2010. Reporting increased in August building to a peak in late October and November before reducing across December and January. The same figure shows that swab positivity for pandemic influenza lags behind the clinical peak by between one to two weeks due to the time delay in testing and reporting. Figure 1A demonstrates that much of the pandemic influenza vaccine administered in the cohort was either at or shortly after the peak in clinical illness presentation.

The number of consultations and consultation rates for the three categories ILI, ILIARI and ARI are summarised in Tables 3 and 4 for both vaccine classification rules used in the statistical models. As all consultations are nested within the ARI category, this has the largest number of consultations followed by ILIARI and then ILI categories. Examining the crude consultation rates for ILI in Table 3, the rate in the unvaccinated group is approximately 4 times that of those vaccinated (0.454 consultations per 1000 person-week (pw) compared to 0.111 consultations per 1000 pw). Splitting the unvaccinated group into those never vaccinated and those individuals who are in the time period before vaccination (Table 4), the consultation rates in those prior to vaccination are slightly higher than those never vaccinated, indicating a possible difference in their consultation behaviour for ILI. Considering the less-specific consultation codes, the difference between the before vaccination and the never vaccinated groups becomes more distinct. Considering ILIARI, when comparing the unvaccinated and vaccinated groups, the consultation rate in the vaccinated group is higher than the unvaccinated group (3.872 per 1000 pw compared to 2.899 per 1000 pw) crudely indicating a negative VE. When looking at the before/never vaccinated split the consultation rate in those prior to vaccination is 6.245 per 1000 pw compared to 2.553 in the never vaccinated group. This indicates that there may be differences in the consultation behaviour of those individuals who seek vaccination and those who do not. This effect is amplified further when the least specific category, ARI is examined. In this case those prior to vaccination have a consultation rate of 11.781 per 1000 pw compared to 2.548 over 1000 pw in those never vaccinated. Examining the unvaccinated and vaccinated split would again imply a negative VE whilst comparing consultations before and after vaccination gives a positive estimate. Such estimation based solely on consultation rates may give misleading results as no adjustment for covariates and the time of consultation is made.

**Table 3:** Consultation numbers and rates for those vaccinated and unvaccinated.

Consultation class	Vaccination status	Person-weeks (pw)	No. of consultations	Consultation rate per 1000 pw
ILI	Unvaccinated	3161068.1	1434	0.454
	Vaccinated	197823.4	22	0.111
ILIARI	Unvaccinated	3161068.1	9163	2.899
	Vaccinated	197823.4	766	3.872
ARI	Unvaccinated	3161068.1	10789	3.413
	Vaccinated	197823.4	1141	5.768

ILI: Influenza-like illness, ILIARI: Influenza-like illness and acute respiratory infections (excluding asthma), ARI: All influenza-like illness and acute respiratory infections (including asthma).

**Table 4:** Consultation numbers and rates for those never vaccinated and those vaccinated before and after vaccination.

Consultation class	Vaccination status	Person-weeks (pw)	No. of consultations	Consultation rate per 1000 pw
ILI	Never vaccinated	2864993.7	1260	0.440
	Before vaccination	296074.4	174	0.587
	After vaccination	197823.4	22	0.111
ILIARI	Never vaccinated	2864993.7	7314	2.553
	Before vaccination	296074.4	1849	6.245
	After vaccination	197823.4	766	3.872
ARI	Never vaccinated	2864993.7	7301	2.548
	Before vaccination	296074.4	3488	11.781
	After vaccination	197823.4	1141	5.768

ILI: Influenza-like illness, ILIARI: Influenza-like illness and acute respiratory infections (excluding asthma), ARI: All influenza-like illness and acute respiratory infections (including asthma).

### Vaccine effectiveness – consultation outcome

Estimates of the adjusted relative risk of a consultation in each of the three consultation outcome categories ILIARI, ILI and ARI, relative to the baseline group, as estimated by the before/after Poisson regression method and Cox Proportional hazards are summarised in Tables 5 and 6 respectively. Estimates for the Poisson model with vaccinated compared to unvaccinated are not presented as they are very similar to the Cox estimates. The summarised VE estimates for all methods are presented in Table 7. Considering the most vaccine specific outcome ILI, the covariate and time adjusted before/after Poisson model results (Table 7) show positive but non statistically significant VE = 34.2% with 95% CI (−0.05, 58.9)% for the pandemic vaccination when comparing vaccinated individuals before and after vaccination. Table 5 shows that receiving seasonal vaccination did not provide any protection with vaccinated individuals shown to be 1.39 times more likely to have an ILI after vaccination. Seasonal vaccination in the previous season had no significant effect. Gender was found to be significant with men 24% less likely to consult for an ILI than women. Young children, aged 0–4 years, have the highest likelihood of consultation and the risk of consultation decreases linearly with age. Individuals with 2 or more ILARI consultations in the previous season were 2.4 times more likely to consult with an ILI. Individuals in a clinical risk group were found to be 1.6 times more likely to consult for an ILI than those not in a clinical risk group. Little effect of deprivation was observed.

**Table 5:** Relative risk of consultation estimated by the Poisson model with before/after split for the three consultation groupings.

		ILIARI		ILI		ARI	
Variable	Level	RR	95% CI	RR	95% CI	RR	95% CI
Pandemic vaccination	After	1.047	(1.077, 0.977)	0.658	(0.411, 1.052)	0.82	(0.758, 0.888)
	Never	0.905	(0.931, 0.877)	1.513	(1.246, 1.839)	0.718	(0.682, 0.756)
Seasonal vaccination	After	0.64	(0.775, 0.709)	1.394	(1.055, 1.842)	0.399	(0.372, 0.428)
	Never	0.43	(0.566, 0.515)	0.766	(0.586, 1.001)	0.214	(0.199, 0.230)
Gender	Male	0.8	(0.769, 0.832)	0.762	(0.687, 0.846)	0.772	(0.744, 0.800)
Age	5–14	0.357	(0.333, 0.383)	0.894	(0.724, 1.103)	0.365	(0.341, 0.390)
	15–44	0.189	(0.178, 0.201)	0.586	(0.483, 0.711)	0.197	(0.185, 0.209)
	45–64	0.171	(0.159, 0.183)	0.312	(0.250, 0.388)	0.148	(0.139, 0.158)
	65–74	0.178	(0.161, 0.197)	0.152	(0.106, 0.217)	0.133	(0.120, 0.146)
	75+	0.154	(0.138, 0.173)	0.042	(0.024, 0.076)	0.106	(0.095, 0.117)
Vaccinated in previous season	Yes	1.382	(1.273, 1.502)	1.398	(1.106, 1.768)	1.319	(1.240, 1.403)
No. of ILIARI in previous season	1	2.579	(2.447, 2.719)	1.708	(1.459, 2.000)	2.219	(2.114, 2.329)
	2+	4.728	(4.438, 5.038)	2.438	(1.923, 3.091)	3.767	(3.549, 3.998)
In clinical risk group	Yes	1.338	(1.249, 1.433)	1.645	(1.387, 1.950)	1.933	(1.823, 2.050)
Carstairs quintile	Q2	1.065	(0.981, 1.157)	1.576	(1.273, 1.951)	1.027	(0.953, 1.106)
	Q3	0.987	(0.921, 1.057)	1.321	(1.094, 1.596)	0.934	(0.877, 0.994)
	Q4	0.976	(0.91, 1.046)	0.981	(0.807, 1.193)	0.983	(0.925, 1.045)
	Q5	1.086	(1.011, 1.168)	1.14	(0.931, 1.395)	0.934	(0.874, 0.998)
	Unknown	1.005	(0.763, 1.324)	1.565	(0.824, 2.972)	1.011	(0.785, 1.301)

Results are relative to the baseline group which is females aged 0–4 in no clinical risk group and in Carstair's deprivation quintile 1 who did not receive a seasonal influenza vaccination in the previous season, had no ILIARI consultations in the previous season, prior to receiving the seasonal influenza vaccination in season 2009/10 and prior to receiving the H1N1 vaccination in season 2009/10 in week 1 (1^st^–7^th^ October). RRs for the specific weeks are not presented. Analysis is based upon consultations up to 31st January 2010.ILI: Influenza-like illness, ILIARI: Influenza-like illness and acute respiratory infections (excluding asthma), ARI: All influenza-like illness and acute respiratory infections (including asthma).

**Table 6:** Hazard ratio of consultation estimated by Cox proportional hazards regression for the three consultation groupings.

		ILIARI		ILI		ARI	
Variable	Level	HR	95% CI	HR	95% CI	HR	95% CI
Pandemic vaccination	Yes	1.128	(1.033, 1.232)	0.507	(0.323, 0.797)	1.044	(0.970, 1.123)
Gender	Male	0.801	(0.770, 0.834)	0.763	(0.687, 0.846)	0.778	(0.750, 0.806)
Age	5–14	0.355	(0.331, 0.380)	0.957	(0.777, 1.179)	0.355	(0.332, 0.379)
	15–44	0.188	(0.177, 0.200)	0.635	(0.524, 0.768)	0.191	(0.180, 0.202)
	45–64	0.172	(0.161, 0.184)	0.339	(0.272, 0.421)	0.152	(0.142, 0.162)
	65–74	0.202	(0.183, 0.223)	0.172	(0.121, 0.244)	0.201	(0.184, 0.220)
	75+	0.174	(0.156, 0.194)	0.048	(0.027, 0.086)	0.155	(0.141, 0.171)
Seasonal vaccination	Yes	0.993	(0.917, 1.075)	1.523	(1.184, 1.960)	0.724	(0.678, 0.773)
Vaccinated in previous season	Yes	1.778	(1.649, 1.916)	1.398	(1.136, 1.721)	2.665	(2.513, 2.826)
No. of ILIARI in previous season	1	2.612	(2.478, 2.754)	1.700	(1.452, 1.990)	2.329	(2.219, 2.444)
	2+	4.809	(4.514, 5.124)	2.411	(1.902, 3.058)	3.995	(3.764, 4.240)
In clinical risk group	Yes	1.524	(1.429, 1.626)	1.565	(1.334, 1.837)	3.113	(2.957, 3.277)
Carstairs quintile	Q2	1.050	(0.967, 1.141)	1.589	(1.284, 1.967)	0.963	(0.895, 1.038)
	Q3	0.970	(0.905, 1.039)	1.319	(1.092, 1.593)	0.868	(0.816, 0.924)
	Q4	0.970	(0.905, 1.039)	0.993	(0.816, 1.207)	0.958	(0.901, 1.019)
	Q5	1.076	(1.001, 1.157)	1.153	(0.942, 1.411)	0.896	(0.839, 0.958)
	Unknown	0.991	(0.752, 1.305)	1.579	(0.831, 2.999)	0.944	(0.733, 1.215)

Results are relative to the baseline group which is females aged 0–4 in no clinical risk group and in Carstairs deprivation quintile 1 who did not receive a seasonal influenza vaccination in the previous season or the current season, had no ILIARI consultations in the previous season and did not receive the H1N1 vaccination in season 2009/10. ILI: Influenza-like illness, ILIARI: Influenza-like illness and acute respiratory infections (excluding asthma), ARI: All influenza-like illness and acute respiratory infections (including asthma).

**Table 7:** VE estimates produced by each of the statistical methods with associated 95% confidence intervals for the three consultation coding groupings: ILIARI, ILI and ARI.

		ILIARI		ILI		ARI	
Adjustment	Method	VE	95% CI	VE	95% CI	VE	
Time only	Poisson before/after	14.1	(5.4, 21.9)	19.1	(−17.5, 50.6)	20.4	(13.9, 26.5)
	Poisson unvaccinated/vaccinated	−71.0	(−84.8, −58.2)	22.3	(−19.7, 49.6)	−121.8	(−136.8, −107.9)
	Cox proportional hazards	−69.4	(−83.1, −56.7)	23.8	(−17.5, 50.6)	−120.2	(−135.0, −106.3)
	Clustered Cox proportional hazards	−69.4	(−98.5, −44.5)	23.8	(−28.6, 54.8)	−120.2	(−152.1, −92.2)
Unadjusted	Screening	34.1	(25.6, 45.5)	20.3	(13.8, 29.8)	46.5	(35.0, 61.8)
Covariate and time adjusted	Poisson before/after	−7.7	(−18.6, 2.3)	34.2	(−0.05, 58.9)	18.0	(11.2, 24.2)
	Poisson unvaccinated/vaccinated	−13.8	(−24.2, −4.2)	48.7	(19.4,67.3)	−5.1	(−13.1, 2.4)
	Cox proportional hazards	−12.8	(−23.2, −3.3)	49.3	(20.3, 67.7)	−4.37	(−12.3, 3.0)
	Clustered Cox proportional hazards	−12.8	(−27.7, 0.3)	49.3	(13.6, 70.2)	−4.37	(−18.5, 8.1)

Analysis is based upon consultations up to 31st January 2010.

Changing the consultation outcome to ARI results in a reduced VE = 18.0% with 95% CI (11.2, 24.2)% and using outcome with asthma codes removed, ILIARI, gives VE = −7.7% (−18.6, 2.3)% (Table 7). The effect of the risk factors for each of these outcomes is broadly similar to those described for ILI consultations apart from individuals with 2 or more consultations in the previous season, which for ILIARI gives RR = 4.728 with 95% CI (4.438, 5.038) which is nearly double the RR observed for ILI consultations and for deprivation.

Detailed results estimated by Cox Proportional Hazards are shown in Table 6. VE results obtained when using Cox Proportional Hazards with the unvaccinated/vaccinated split and Poisson regression with the same split were broadly similar (Table 7). Examining the Cox Proportional Hazards results in detail, the effect of the individual risk factors across the consultation categories is similar to the effects observed in the before/after Poisson model. There are however some exceptions. For the ILIARI and ARI groupings, both being in a clinical risk group and receiving the pandemic vaccination in the previous season have inflated RRs compared to the before/after Poisson model (Table 5 and 6). Considering ARI in particular, the RR for receiving seasonal influenza vaccination in the previous season increased from 1.319 to 2.655 and for clinical risk group increased from 1.933 to 3.113. The strength of these adjustments is reflected in the substantial differential between the unadjusted and adjusted models when using Cox Proportional Hazards and Poisson regression with unvaccinated/vaccinated split methods as presented in Table 7 for the ILIARI and ARI consultation groupings.

The VE estimates obtained using the screening approach, which mirrors a situation where only aggregate end of season data is available and therefore makes no adjustment for confounders and temporal effects, is summarised in Table 7. The estimates from the screening method for ILI are consistent with the time-adjusted models. For the less specific consultation outcomes of ILIARI and ARI the VE estimates are higher than both the time-adjusted and the time and covariate adjusted models.

**Figure 3:**
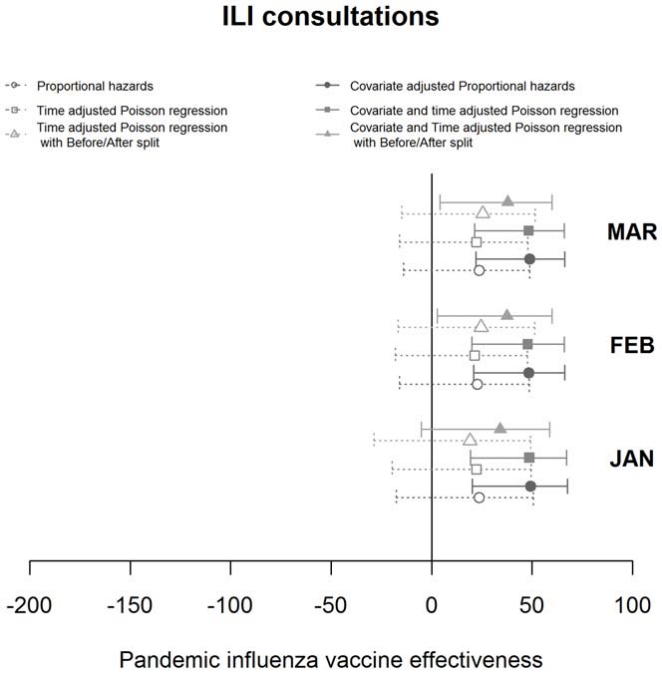
Graphical representation of the VE estimates produced by month by each of the statistical models with associated 95% confidence intervals for the ILI consultation grouping.

**Figure 4:**
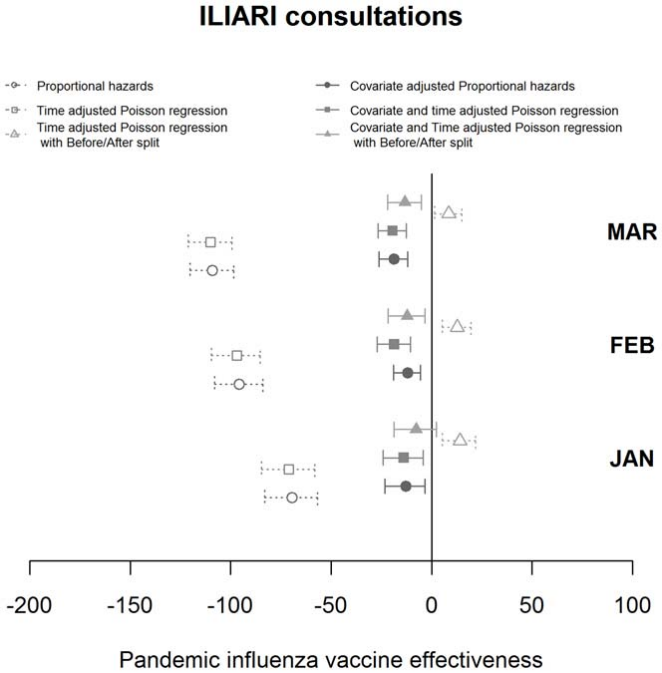
Graphical representation of the VE estimates produced by month by each of the statistical models with associated 95% confidence intervals for the ILIARI consultation grouping.

**Figure 5:**
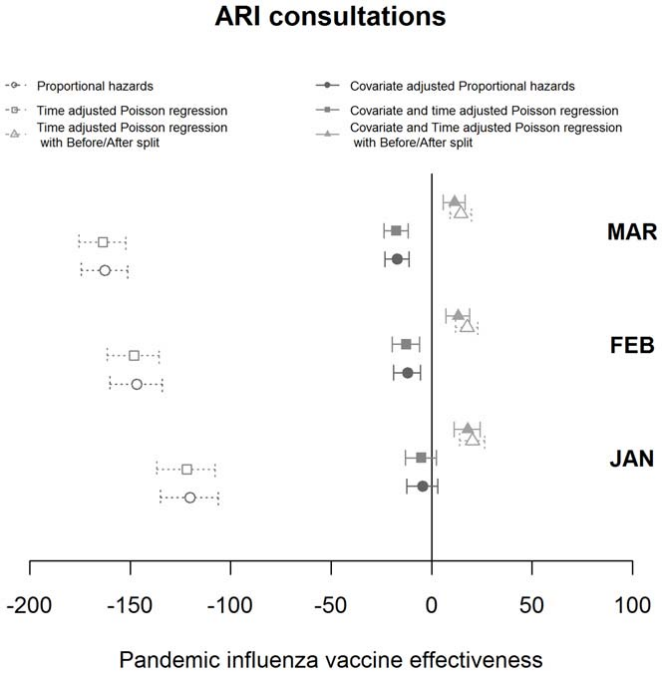
Graphical representation of the VE estimates produced by month by each of the statistical models with associated 95% confidence intervals for the ARI consultation grouping.

Estimates of VE for each consultation class and statistical model used, varying by the cut-off date for analysis, are shown in Figures 3, 4 and 5. The pattern observed between the estimates achieved with differing methods varied with the consultation class analysed. Results for the ILI consultation (Figure 3) class gave comparable results for the Poisson and Cox Proportional hazards models but results around 10 percentage points lower using the Poisson before/after approach. The ILI response, being most specific, was relatively invariant to the time point used as an endpoint. For ILIARI (Figure 4) and ARI (Figure 5) consultation categories there was variation in the estimates found dependent on whether adjustment was performed, particularly for the Poisson and Cox Proportional hazards models. For ARI consultations there is more variability in the VE estimates with the cut-off date used for analysis. The point estimates of VE decrease in February and again in March. As ARI is less specific to influenza, this decrease is related to the levels of influenza circulating in the community and how these levels are relative to other respiratory pathogens. Figure 2 illustrates that influenza levels peaked in November, when 45% of swabs tested positive and decreased to less than 5% positive by the end of January. Post January, only 3 further cases were virologically confirmed with the last being found on 14^th^ March. This indicated the lack of influenza circulating in February and March and a higher proportion of ARI consultations in this time may be attributable to other respiratory pathogens to which the vaccination would offer no protection.

The effect of accounting for the clustering of individuals within practices, using robust standard errors with the Cox approach (Table 7), widens the variability surrounding the estimates of VE but any significant overall vaccine effect found under the original Cox approach remains.

### Vaccine effectiveness – virology outcome

Of those individuals in the cohort, a total of 1711, were swabbed, corresponding to 8.9% of the cohort. This is a high proportion of the swab test results (12.6% of 13623 tests) as the GP practices in PIPeR are all in the GP sentinel surveillance scheme. The associations between consultations for ILIARI during the season and having a virological test are shown in Table 1. These show that 50% of the cohort is female, 57% of those who consulted for an ILIARI are female and the 58% of those (with symptoms) tested are female. Thus there is no selection bias for a virological test based upon gender. The major bias is associated with Age where there is over representation, compared to consultations, among those swabbed in the 15–44 age group and under representation among children aged under 5 and adults aged 65 and over. There is also a bias associated with deprivation in that patients in a more deprived neighbourhood are more likely to be swabbed, but no bias associated with risk group membership, seasonal vaccination in the previous year and number of consultation for ILIARI in the previous year.

A total of 508 tested positive for H1N1v influenza, yielding positivity rate of 29.7%. The majority of these patients were unvaccinated at the time of swabbing (1657); and only 54 were swabbed post vaccination. Among those not vaccinated, swab positivity is higher among those in a risk group (403 positive, 920 negative) compared to those not in a risk group (102 positive, 232 negative), *p* = 0.002. Very few vaccinated patients were tested −54 patients and only 3 were positive for H1N1v. Trends in swab positivity are presented in Figure 1 where it is seen that positivity peaked in October 2009 and decreased over time among those unvaccinated.

The results of fitting the logistic regression with a quadratic temporal trend in week to swab positivity results are presented in Table 8. Alternative means of controlling for the temporal trend were considered - a spline trend within a generalised additive model for the weekly temporal trend and a factor with 4 levels representing the 4 four week period. The parameter estimates were relatively unaffected by the method of estimating the trend and the estimates from the most parsimonious model using a quadratic trend are discussed. Adjusting for the other factors in the model there is no evidence of any effect on swab positivity of seasonal vaccination status, risk group, deprivation and gender. There are trends with Age group, p<0.0001, with greater swab positivity among those aged 5–14 and 15–44 years. There was no evidence of any interactions with vaccination status and similar results were obtained for the vaccine effects when considering only those in a risk group. Relative to those who were unvaccinated at the time of swabbing the odds ratio of testing positive is 0.40 (95% CI 0.11, 1.38), *p* = 0.17. This corresponds to a vaccine effect of 60% (95% CI −38%, 89%).

**Table 8:** Odds ratios and 95% confidence intervals for the effects of the listed factors on H1N1v swab positivity among patients under 65 who were swabbed as part of the Scottish Sentinel Swabbing Scheme.

		GAM			Quadratic			Four Week Factor		
Factor	Level	OR	95% CI	P	OR	95% CI	P	OR	95% CI	P
Pandemic Vaccine	No	1.00	-		1.00	-		1.00	-	
	Yes	0.42	(0.12, 1.46)	0.170	0.40	(0.11, 1.38)	0.145	0.39	(0.11, 1.36)	0.139
Seasonal Vaccine	No	1.00	-		1.00	-		1.00	-	
	Yes	1.19	(0.70, 2.02)	0.520	1.19	(0.70, 2.02)	0.521	1.14	(0.67, 1.93)	0.635
Risk Group	No	1.00	-		1.00	-		1.00	-	
	Yes	0.84	(0.62, 1.13)	0.256	0.85	(0.63, 1.14)	0.272	0.86	(0.64, 1.15)	0.305
Age Group	<1	1.00	-		1.00	-		1.00	-	
	1–4	2.05	(0.24, 17.65)	0.514	2.11	(0.25, 18.12)	0.496	2.12	(0.25, 18.10)	0.493
	5–14	7.38	(0.87, 62.87)	0.068	7.61	(0.90, 64.58)	0.063	7.60	(0.90, 64.30)	0.063
	15–44	3.72	(0.44, 31.53)	0.228	3.87	(0.46, 32.66)	0.213	3.87	(0.46, 32.52)	0.213
	45–64	2.05	(0.24, 17.65)	0.512	2.13	(0.25, 18.24)	0.490	2.13	(0.25, 18.14)	0.490
	65–74	0.70	(0.07, 6.97)	0.761	0.72	(0.07, 7.13)	0.778	0.74	(0.08, 7.34)	0.799
	75+	0.29	(0.02, 3.83)	0.350	0.31	(0.02, 4.07)	0.375	0.31	(0.02, 4.06)	0.374
Gender	Female	1.00	-		1.00	-		1.00	-	
	Male	1.01	(0.80, 1.27)	0.960	1.00	(0.79, 1.26)	0.999	1.01	(0.80, 1.27)	0.945
Carstairs Quintile (Deprivation)	Q1	1.00	-		1.00	-		1.00	-	
	Q2	1.30	(0.77, 2.18)	0.323	1.29	(0.77, 2.16)	0.336	1.25	(0.75, 2.09)	0.395
	Q3	1.43	(0.90, 2.29)	0.132	1.42	(0.89, 2.27)	0.140	1.40	(0.88, 2.24)	0.157
	Q4	1.20	(0.76, 1.92)	0.435	1.20	(0.75, 1.91)	0.445	1.20	(0.75, 1.92)	0.438
	Q5	1.31	(0.83, 2.08)	0.244	1.30	(0.82, 2.06)	0.262	1.29	(0.81, 2.03)	0.281
	Unknown	1.44	(0.43, 4.87)	0.557	1.46	(0.44, 4.90)	0.540	1.48	(0.44, 4.94)	0.527

The swabs were all collected between October 1^st^ 2009 and 31^st^ January 2010. Three different models were used to control for the temporal trend – a spline trend within a generalised additive model (GAM) for the weekly temporal trend; a quadratic trend; and a factor with 4 levels representing the 4 four week period.

### Vaccine effectiveness – death outcome

A total of 623 patients are recorded on the GP systems as having died in the 4 month study period giving a crude death rate of 9.8 per 1000 population per year (95% CI 9.0, 10.5) compared to the Scottish rate of 10.5 per 1000 population per year. This suggests under reporting of deaths on the GP systems.

The parameter estimates from the Cox regression model are presented in Table 9. Pandemic vaccine is associated with a reduction in the risk of death with a relative risk of 0.60, (95% CI 0.44, 0.82) and this is similar to the relative risk associated with the use of seasonal flu vaccine 0.52 (95% CI 0.42, 0.64). Temporal changes in the vaccine effects for the death outcome are presented in Table 10. The H1N1v vaccine effect clearly wanes over time and once the pandemic has ceased there is no vaccine effect on mortality. For seasonal influenza vaccination the pattern is similar though the effects persist during the winter season but once the pandemic H1N1v activity has ceased.

**Table 9:** Hazard ratios of death, with 95% confidence limits, estimated by Cox proportional hazards regression.

Factor	Level	HR	95% CI	P-value
Pandemic Vaccine	No	1.00	-	
	Yes	0.60	(0.44, 0.82)	0.001
Seasonal Vaccine	No	1.00	-	
	Yes	0.52	(0.42, 0.64)	0.000
Seasonal Vaccine	No	1.00	-	
Previous Season	Yes	1.45	(1.18, 1.78)	0.000
Number of ILIARI consultations in previous year	0	1.00	-	
	1	1.19	(0.89, 1.58)	0.251
	2	1.12	(0.60, 2.09)	0.732
	3+	2.74	(1.61, 4.66)	0.000
Risk Group	No	1.00	-	
	Yes	4.96	(3.99, 6.17)	0.000
Age Group	<14	1.00	-	
	15–44	2.44	(0.73, 8.18)	0.149
	45–64	11.39	(3.59, 36.13)	0.000
	65–74	77.75	(24.51, 246.62)	0.000
	75+	355.21	(112.98, 1116.77)	0.000
Gender	Female	1.00	-	
	Male	1.25	(1.06, 1.46)	0.007
Carstairs Quintile (Deprivation)	Q1	1.00	-	
	Q2	0.99	(0.70, 1.40)	0.945
	Q3	0.85	(0.63, 1.15)	0.290
	Q4	1.01	(0.75, 1.36)	0.964
	Q5	1.10	(0.81, 1.51)	0.536
	Unknown	1.30	(0.47, 3.60)	0.611

**Table 10:** Vaccine effect on overall mortality estimated by Poisson regression in various time periods after the beginning of the study.

Start	End	Epidemic period	Pandemic H1N1v Vaccine			Seasonal Vaccine		
			VE	95% CI	P-value	VE	95% CI	P-value
01/10/2009	25/11/2009	Peak	100.0	-	-	71.6	(57.0, 81.3)	0.000
26/11/2009	23/12/2009	Wane	62.4	(24.6, 81.2)	0.006	49.1	(23.2, 66.3)	0.001
24/12/2009	20/01/2010	Wane	38.3	(8.2, 58.5)	0.017	18.7	(−22.4, 46.0)	0.321
21/01/2010	17/02/2010	After	30.8	(−3.3, 53.6)	0.072	52.2	(27.0, 68.6)	0.001
18/02/2010	17/03/2010	After	−0.9	(−48.3, 31.3)	0.963	24.5	(−20.7, 52.8)	0.240
18/03/2010	30/04/2010	After	1.7	(−36.1, 29.0)	0.918	−15.7	(−75.6, 23.8)	0.495

In the first period from 1/10/2009 to 25/11/2009 no pandemic H1N1v vaccinated individuals died hence the VE is estimated as 100% and no confidence intervals supplied.

## Discussion

This study has provided vaccine effect estimates for the pandemic influenza A H1N1v vaccine for consultations, virology and death. Comparing unvaccinated with vaccinated the vaccine effect was 60% (95% CI −38%, 89%) for virology confirmed influenza cases, among patients with symptoms, 49% (95% CI 19%, 67%) for ILI consultations, and 40% (95% CI 18%, 56%) for overall mortality; the latter two among all patients in the cohort. There was no significant vaccine effect for ILIARI and ARI consultations with negative estimates. The virology estimate was not significant and not inconsistent with those based upon the test negative design, [2]–[5], though it is towards the lower limit of the confidence intervals. The low number of vaccinated individuals who are swabbed is the main reason for the imprecision in the estimate.

In this study the results about the effect of the seasonal influenza vaccine are conflicting. The analysis of ILI consultations showed that patients who received the seasonal flu vaccine in the current season had a 53% increase in consultations (95% CI 18%, 96%) this slightly greater than the effect of receiving seasonal vaccine in the previous year, 40% (95% CI 14%, 72%). This result is consistent with the increased risk of medically attended ILI reported by Skowronski *et al.* [14], however the virology analysis does not confirm this as it shows a non-significant 19% increase. The consultation rates for ILIARI and ARI are reduced among patients who received the seasonal flu vaccine.

The vaccine effectiveness estimate varies dependent on the group of clinical codes used within the cohort for the description of their respiratory infection. Thus a different answer on effectiveness is obtained dependent on whether we chose just influenza like illness (ILI) or all acute respiratory infections (including influenza like illness) (ARI) and whether we include or exclude asthma exacerbations within this category (ILIARI). Although ILI conditions alone may be more specific, the number of observations in the PIPeR cohort are relatively small hence the VE estimate and confidence intervals around any estimate vary widely for a long period before a more precise estimate is obtained. For ILIARI we have a greater number of observations and this addresses the problem of variability in general practice recording, whereby there may be failure to record ILI as a diagnosis, particularly in young children or individuals with milder disease manifestation. However, the lower specificity of the ILIARI code grouping may lead to underestimation of the vaccine effectiveness estimate. In the adjusted models we present, the less specific ILIARI code grouping consistently produced the lowest VE estimate.

Using the ARI grouping, the VE estimate obtained by all methods is greater than the estimate obtained for ILIARI, which differs only by excluding asthma codes. Whilst a range of respiratory pathogens can induce exacerbations of asthma, influenza is a potent precipitant of asthma episodes either by itself or as a consequence of co-infection with other respiratory viruses or by virtue of secondary bacterial infections that may follow the initial influenza infection. This is particularly so in younger people. The finding of a further reduction in clinical presentations after vaccination from the Poisson regression model comparing before vaccination with after vaccination, leading to an increased VE estimate when all acute respiratory infections including asthma are measured, is then an expected finding. There are, however interpretational difficulties associated with differentiating between consultations associated with environmental challenges rather than infective challenges in asthma exacerbations. Therefore the estimate of VE may be inflated artificially by inclusion of asthma codes. It should also be noted that, the specificity of the ARI and ILIARI categories will be influenced by the incidence of influenza relative to other circulating respiratory illness and therefore using ILI consultations for estimations is the preferred choice. In non-pandemic years, there has however been a lack of recording of the class despite influenza circulating. It is therefore essential that other groupings are available for analysis.

The vaccine effectiveness estimate was also found to depend on the statistical methodology used. For ILI the estimates were similar however adjustment for confounding variables and propensity to consult had a larger effect for the two methods based upon the comparison of vaccinated with unvaccinated. This is because the vaccine effect is essentially a between person estimate in these models whereas in the before vaccination after vaccination framework there is a matching with the same individuals in both groups. The before vaccination after vaccination framework method should produce more precise results by directly accounting for possible differences in the propensity to consult between the vaccinated and unvaccinated individuals though will not do so if the propensity changes post vaccination. Furthermore, the before after framework is retrospective, in that it is vaccine status at the end of the season which determines the exposure group and this is not as statistically valid as the prospective framework for the time dependent Cox model or Poisson Regression model.

For both ILIARI and ARI there is a large change in the vaccine effect estimates associated with adjustment for confounding from the two methods comparing vaccinated and unvaccinated patients. For all three methods the adjustment serves to increase the estimated vaccine effect.

The more simplistic screening method, based on aggregate data and unadjusted for time and covariates, gave similar results to the other methods for ILI but for the less specific consultation groupings gave over estimates of the effectiveness. This is likely due to the lack of adjustment the healthy vaccine effect and the change in consultation patterns across the time frame considered.

Linking the cohort to all virology tests in Scotland has shown some of the biases in having a swab and it was demonstrated that patients aged 15–44 who had symptoms were more likely to be swabbed that older and younger symptomatic patients. It was demonstrated that having a swab was unrelated to gender, membership of a risk group, having had the seasonal vaccination in the previous year and number of consultations in the previous year. Other than age the only other determinant of swabbing was deprivation and this is may be associated with swabbing frequency in the general practices.

Using death as an endpoint yields results which are difficult to interpret as both the pandemic vaccine and the seasonal vaccine are independently associated with a reduction in the risk of death. During the 2009–10 pandemic season the confirmed death rate from Influenza A H1N1v was low and a 40% reduction in death rate from influenza vaccine is rather large – however this is a relative reduction in mortality. We cannot discount, however, that the effect is not a ‘healthy vaccinate effect’ in that frail patients were not less likely to be vaccinated and this would certainly explain both the pandemic and seasonal vaccine effects. The trends over time in this effect show that the effects are present during the winter period when there are traditionally higher death rates, particularly among those over 65.

Overall, this analysis has provides support for an association between influenza and increased consultations. The low levels of VE may be related in part to the fact that Influenza A H1N1v activity had begun to wane by the time vaccine was distributed. Similar estimates of vaccine effect have been found using different methodologies but the results are found to vary with the measure of outcome used. Understanding the variation in the estimates between the methods used and the outcome considered aids analysis and interpretation of vaccine effectiveness results in future seasons.
